# A longitudinal investigation of mental health, perceived learning environment and burdens in a cohort of first-year German medical students’ before and during the COVID-19 ‘new normal’

**DOI:** 10.1186/s12909-021-02798-2

**Published:** 2021-08-02

**Authors:** Ann-Kathrin Schindler, Sabine Polujanski, Thomas Rotthoff

**Affiliations:** Medical Didactics and Educational Research; DEMEDA (Department of Medical Education); Medical Faculty, University of Augsburg, Universitätsstr. 2, 86159 Augsburg, Germany

**Keywords:** Mental health, New normal, First-year medical students, Undergraduate medical students, COVID-19, Well-being, Burnout, Depression, Learning environment, Virtual learning

## Abstract

**Background:**

Medical students’ propensity to develop mental morbidity has been described for decades but remains unresolved. To assess student mental health person-centred and longitudinally, we have been investigating a cohort of German students since October 2019. After their first semester under ‘normal’ conditions, rapid changes became necessary due to the COVID-19 situation. In line with the initial aim, we investigated students’ change of mental health, perceived learning environment and burdens in the ‘new normal’.

**Methods:**

Students in a newly founded German medical study programme (*n* = 63) answered a questionnaire each semester (October 2019 = entering medical school; December 2019 = ‘old normal’; June 2020 = ‘new normal’; December 2020 = ‘new normal’) on their well-being (FAHW-12), burnout (Maslach Inventory), depression (PHQ-9), perception of the learning environment (DREEM), burdens and protective attitudes in the ‘new normal’ (items designed for the study).

**Results:**

Friedman tests reveal overall significant differences (all *p <* .001*)* in depression and burnout (emotional exhaustion, depersonalisation, personal accomplishment); changes in well-being were identified as just non-significant (*p =* .05). The effects were explained by a significant increase in burnout and depression identified post-hoc from October 2019 to December 2019. No increase in severity was identified in the ‘new normal’ semesters. The learning environment was perceived positively even with a significant improvement for June 2020 (repeated measures ANOVA *p* < .001). Study-related burdens (e.g. procrastination of online-learning material) took on greater relevance than burdens related to physicians’ occupation (e.g. potential for students' recruitment to the healthcare system during their studies).

**Conclusions:**

The ‘new’ when entering medical school had a greater impact on our students’ mental health than the ‘new normal’. The readiness for change in the context of a newly designed study programme may have been beneficial with regard to students’ positively perceived learning environment during the virtual semesters. Monitoring medical students’ mental health longitudinally should be a concern regardless of a pandemic.

## Introduction

Medical students’ propensity to develop mental morbidity in the course of their study has been described for decades but remains unresolved [1]. A US survey revealed a significantly higher prevalence of burnout and depressive symptoms for medical students in comparison to other US college graduates. In their sample of about 4000 medical students across various years in training, 50% reported critical burnout scores (measured by emotional exhaustion), and 58% reported depressive symptoms. In a reference group of about 700 college students, 32% reported critical burnout values and 48% reported depressive symptoms [2]. A meta-analysis identified a range of 9 to 56% of medical students suffering from depression [3]. This is problematic for entering the occupation as a physician in the future [3]. Physicians’ mental morbidity is known to increase mistakes, risk patients’ safety and cause staff shortage and, therefore, economic damage [4].

Slavin [1] states reasons why this long-lasting problem remains unsolved: 1) the conviction that a tough study programme prepares for a tough occupation; 2) underestimation of prevention rather than treatment of mental morbidity; 3) a focus on curricular changes on the implementation of new content and teaching methods rather than the prevention of students' mental health; 4) the association of unfavourable students’ mental health conditions with poor institutional quality; and 5) a focus on the individual self-care level rather than on the learning environment—as a systematic problem.

Based on these demands, we started a longitudinal study in October 2019 monitoring medical students’ initial mental health and afterwards each semester throughout their entire study programme. Our sample was withdrawn from the youngest German medical faculty, which sets up a new, reform-based curriculum. Reform-based means the implementation of a competency-based curriculum based on the (German) National Catalogue of Competence-Based Learning Objectives for Medical Education [NKLM].^1^ The NKLM states physicians’ (mental) health is a compulsory learning goal, e.g. ‘Graduate students are able to explain physical and mental stress and consider individual resilience in medical care’. The obtained data will be applied to address demands 1–5 [1] in curricular developments, revisions and adjustments.

After the first semester under ‘normal’ conditions, faculty developers, teachers and students were additionally challenged by the rapid changes necessary due to the COVID-19 pandemic, which has continued until now and will continue at least to the forthcoming semester. This ‘new normal’ provokes (digital) chances but also potential burdens because of certain implications that would not have been transpired at such tremendous speed [5–8]. The need for psychological care from educational institutions during a crisis [7] and attention to trainees’ wellness and monitoring of burnout in the disrupted or restricted training situation due to COVID-19 [9] is emphasized. This demand fit very well with our concurrently conducted longitudinal study. In this paper, we sought 1) to investigate students’ change and readjustment perceptions to the ‘new normal’ and 2) to discuss the role of the study-status and study-context as well as the country’s pandemic situation when interpreting the data.

Concretely, we addressed the following research question: *How does a cohort of first-year German medical students in a reform-based study programme perceive their ‘new normal’ with regard to changes in mental health, perceived learning environment and burdens?*

## Study status and context

### Study status: entering medical school

Even outside of a pandemic, for a first-year medical student, the sensation of ‘new’ surpasses ‘normal’ with regard to literally every aspect of campus life: study routines, peers, etc. Heinen and colleagues [10] found a higher stress level (operationalised by tension, worry and demands) for first-year medical students: a) in comparison to an age-specific German student norm and b) in comparison to a second-year cohort (they referred to another study conducted by Fliege et al. [11]). The authors emphasised the life-changing processes that take place when entering university, such as developing new relationships and getting used to university learning and assessment practices. Undergraduate medical students report academic workload as one major stressor [12]. For medical students, reflections on their intended occupation concerning suffering, illness and death can be additional stressors—more obvious in the pandemic. For *first-year* students, the experience of growing into a collaborative, campus-based study experience [13]—with intense academic expectations—is turned upside-down in a continuing virtual study situation. Additionally, the disruption of study and learning activities can become a relevant stressor because it can cause uncertainty about completing requirements [14].

### Study context: medical education in times of COVID-19

The COVID-19 pandemic has brought drastic discrepancies in the country’s healthcare systems to the fore [5]. Within the balancing act of maintaining safe patient care and fulfilling the responsibility of educating future physicians [15], trainees’ roles have varied from recruitment to the healthcare frontlines to complete exclusion from any on-campus learning activity [16]. Survey data [17] reveal the COVID-19 pandemic’s impact on final-year medical students in the United Kingdom: On the one side, the majority of students claimed a reduced feeling of preparedness for beginning their work as a physician because of practicum interruptions. On the other side, students reported a high willingness to join the workforce to support the pandemic.

Additionally, economically dependent technical equipment, prior experience and acceptance of virtual learning might vary drastically among educational institutions. German colleagues [18] questioned higher-education students of various faculties (including a sample of 280 medical students) on their digital readiness, revealing high overall self-reported digital learning skills and satisfactory equipment. Additionally, better equipment, longer e-learning experiences and higher self-reported skills in digital learning were accompanied by reduced stress (tension, overload, worries and loneliness) and a better work–life balance.

## Methods

### A new, reform-based programme as the study context

As stated in the introduction, our sample is situated in a small, reform-based medical study programme currently in an ongoing intense curriculum-development process. The curriculum intends an intense connection of biomedical (pre-clinical) basics, clinical subjects, medical skills and clinical and research experience. During the first two years, students are predominantly involved in lectures, seminars, tutorial groups and skills training, but they also become engaged in bed-side teaching and first clinical insights. In the ‘new normal’ semesters, teaching formats became virtual, and skills-training and bed-side teaching only took place if warranted by the pandemic situation.

The curriculum had encompassed readiness for innovation in the effort to design a blended-learning curriculum before the online semester. Students had used online sessions to prepare and follow-up the lectures and seminars; thus, the e-learning platform Moodle had been familiar to students and lecturers during their first semester (October 2019–March 2020). For the transformation to ‘online-teaching’, lecturers perceived a high level of support by the medical didactics team in the interactive application of Zoom for (predominantly applied) synchronous virtual teaching. Lecturers were supported in providing e-learning beyond material supply.

### Design

The study was conducted in the first cohort of medical students (84 students registered in October 2019) in the study programme. Students answered survey questionnaires (each 30 min) in

- October 2019 about entering medical school (paper format).
- December 2019 about the first semester in the ‘old normal’ (paper format).
- June 2020 about the second semester in the ‘new normal’ (online format).
- December 2020 about the third semester in the ‘new normal’ (online format).

There were 80 students who gave their informed consent to the voluntary longitudinal questioning without incentive. An information document was sent to the students the week before the first survey. Only datasets of students (all participants were > 18 years old) who gave an informed paper-based consent were included in the data analysis. For the paper-based measurement points, missing values were completed by the instrument-specific procedure. Online, all items had to be answered to avoid missing values.

**Fig. 1:**
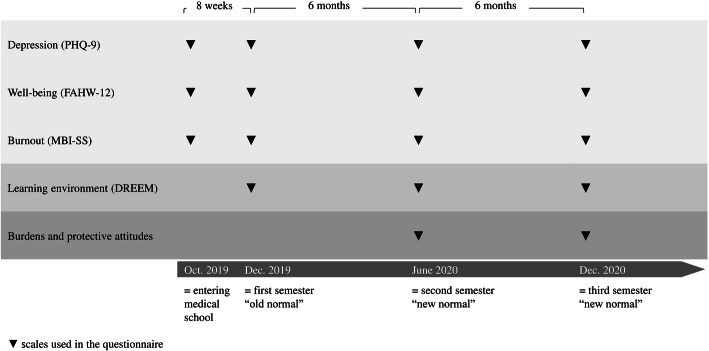
Applied instruments at the four measurement points

As illustrated in Fig. 1, mental health constructs were assessed at all four measurement points. Students rated the learning environment for December 2019, June 2020 and December 2020. In October 2019 students were new to the study programme and could not judge the learning environment. In the virtual semesters (June 2020 and December 2020), students answered items on their burdens and protective attitudes associated with the COVID-19 situation. The detailed instruments are described in the following section.

### Instruments

All instruments showed acceptable reliability (α_all instruments_ > .70) and are described in the following:

*Depression* was investigated by nine items of the PHQ-9 (depression module of the PHQ-D Patient Health Care screening instrument to identify the severity of mental disorders) on a four-point Likert scale (0 = not at all; 3 = nearly every day). The maximum sum-score is 27. An example item is: ‘Over the last four weeks, how often have you been bothered by any of the following problems? … *Little interest or pleasure in doing things*’ [19] (German version [20]).

*Well-being* was assessed by the 12 items of the FAHW-12 (original instrument in German [21]) on a five-point Likert scale (1 = certainly not; 5 = yes, exactly like that). Six items measured well-being (e.g. ‘I am very balanced’) and six items measured the lack of well-being (e.g. ‘I can no longer stand the inner tensions’). The maximal sum-score of 25 was calculated by the following procedure: (the six items of well-being × 5 points (total agreement)) – (the six items of lack of well-being × 1 point (total disagreement)).

*Burnout* was assessed on three subscales (three items of *emotional exhaustion*; three items of *depersonalisation*; three items of *personal accomplishment*) of the Maslach inventory (seven-point Likert scale 0 = never; 6 = daily; mean-score instrument). *Emotional exhaustion* referred to the physical condition of the students, i.e., to what extent they feel drained and fatigued by their studies (example item: ‘I feel emotionally drained by my studies’). *Depersonalisation* referred to changes in enthusiasm and interest in studying (example item: ‘I have become less enthusiastic about my studies’). *Personal accomplishment* measured how students assessed their own professional competence and performance (example item: ‘During class I feel confident that I am effective in getting things done’) [22, 23] (German version [24]).

*Learning environment* was assessed by the 50 items of the Dundee Ready Education Environment Measure (DREEM) on a five-point Likert Scale (0 = I do not agree at all; 4 = I totally agree). The maximal sum score is 200. DREEM assessed the students’ perception of teaching (12 items, e.g. ‘The teaching is well focused’); perception of teachers (11 items, e.g. ‘The teachers are well prepared for their classes’); perception of atmosphere (12 items, e.g. ‘I feel able to ask the questions I want’); academic self-perceptions (8 items; e.g. ‘I am confident about passing this year’); and social perceptions (7 items, e.g. ‘My social life is good’) [25, 26] (German version [27]).

For the questionnaires applied during the ‘new normal’ semesters in June 2020 and December 2020, we designed items on students’ perceived *burdens* on a five-point Likert scale (0 = fully disagree; 4 = fully agree). These were inspired by the *JAMA* viewpoint published in March 2020 [6], which was dedicated to medical education during the COVID-19 pandemic. Three items assessed *burdens related to physicians’ occupation* (e.g. ‘Pursuing a profession with risk to personal health’). By these three items, we intended to investigate whether medical students—as trainees in the healthcare system—felt challenged by the pandemic. Four items questioned students on their *study-related burdens* (e.g. ‘Procrastination of learning the online-learning material’) and two items questioned them on *burdens related to their personal situation* (e.g. ‘My living situation (e.g. quiet study place)’) during the virtual semesters. In addition to the burdens, we asked students on protective attitudes in their role as medical students (e.g. ‘My medical knowledge and interest are an advantage in dealing with the COVID-19 situation’) on the same five-point Likert scale as their burdens.

### Analysis

Using SPSS26, we analysed the longitudinal data (two-sided; 95% CI) with Friedman-tests (ordinal scale level and restricted normal distributions), including Dunn–Bonferroni post-hoc testing. The interval scale level and normal distribution (*p*_Shapiro-Wilk_ > .05) for the learning environment allowed for a repeated measures ANOVA. The reported effect sizes for Friedman-test-related results were: Pearson’s *r* (*r* = .1, small effect; *r* = .3, medium effect; *r* = .5, large effect) and Cohen’s η^2^ (η^2^ = .01, small effect; η^2^ = .06, medium effect; η^2^ = .14, large effect) for ANOVA results [28]. Students’ burdens and attitudes during the ‘new normal’ semesters (June 2020 and December 2020 measurement points) were illustrated in histograms and tested by Wilcoxon-tests for differences between the two measurement points. Pearson’s *r* is the reported effect size.

### Ethics approval

Methods were carried out in accordance with the Declaration of Helsinki [29] and the European Data Protection Law [30]. Study protocols, instruments and consent documents were approved by the data-protection supervisor and head of the ethics committee of the University of Augsburg, who excluded ethical concerns of any kind (negative clearance certificate 4 October 2019).

## Results

There were 63 students (response rate 79%; female 63%; mean age_study start Oct 2019_ = 21.05 years, SD = 4.5) who answered questionnaires on all four measurement points.

### Mental health

We identified significant overall differences (Table 1) for all mental health constructs with large effects related to depression (*r* = .61), emotional exhaustion (*r* > .99), depersonalisation (*r* = .73) and personal accomplishment (*r* = .84). Overall changes of well-being were identified as just non-significant (*p =* .05); thus, no further post-hoc testing was applied. For the depression and burnout constructs, post-hoc testing revealed October 2019 as the relevant point of reference for the described effects. In the following, we explain the descriptive and post-hoc results.

### Depression

 Students reported just ‘mild depression’ for October 2019 (median = 5.00; IQR = 3.0–7.0) which—within the level of ‘mild depression’—intensified significantly in December 2019 (median = 7.00; IQR = 4.0–11.0). During the ‘new normal’ semesters, values remained at a comparable level in June 2020 (median = 7.00; IQR = 5.0–10.0) and December 2020 (median = 7.00; IRQ = 4.0–10.0).

**Table 1:** Longitudinal results of medical students’ mental health and perceived learning environment

						Overall		Post-hoc											
		Oct. 2019	Dec. 2019	June 2020	Dec. 2020			Oct 19– Dec 19		Oct 19–June 20		Oct 19– Dec 20		Dec 19–June 20		Dec 19– Dec 20		June 20– Dec 20	
	Max Scoring	median (IQR ^e^)	median (IQR ^e^)	median (IQR ^e^)	median (IQR ^e^)	*p*	*r* ^f^	*p*	*r* ^f^	*p*	*r* ^f^	*p*	*r* ^f^	*p*	*r* ^f^	*p*	*r* ^f^	*p*	*r* ^f^
Depression ^a^	27	5.0 (3.0–7.0)	7.0 (4.0–11.0)	7.0 (5.0–10.0)	7.0 (4.0–10.0)	<.001	.61	.001	.47	<.001	.50	.005	.42	>.99	–	>.99	–	>.99	–
Well-being ^b^	25	12.0 (8.0–17.0)	9.0 (5.0–14.0)	11.0 (4.0–15.0)	12.0 (4.0–16.0)	.05	.35												
Burnout – Emotional exhaustion ^c^	6	1.33 (0.67–2.00)	3.0 (2.0–4.0)	3.0 (2.33–4.0)	2.67 (1.67–3.67)	<.001	>.99	<.001	.89	<.001	.96	<.001	.69	>.99	–	.63	–	.18	–
Burnout – Personal accomplishment ^c^	6	0.67 (0.0–1.33)	1.67 (1.0–3.0)	2.0 (1.0–3.0)	1.33 (0.67–2.33)	<.001	.84	<.001	.70	<.001	.67	.004	.42	.72	–	.18	–	.37	–
Burnout – Depersonalisation ^c^	6	0.0 (0.0–0.0)	0.33 (0.0–1.0)	0.67 (0.0–1.67)	0.33 (0.0–1.0)	<.001	.73	.01	.40	<.001	.57	.004	.43	>.99	–	>.99	–	>.99	–
			mean (SD)	mean (SD)	mean (SD)	*p*	η^2 g^							*p*		*p*		*p*	
Learning environment ^d^	200		128.2 (21.4)	126.2 (22.5)	134.5 (18.2)	<.001	.14	-- ^h^	–	–	–	–	–	.90	–	.01		<.001	

^a^ Sum-score instrument: 0–4 no depression; 5–9 mild depression; 10–14 moderate depression; 15–19 moderately severe depression; 20–27 severe depression

^b^ Sum-score instrument: 0–5 severely below average; 6–9 below average; 10–13 average; 14–16 above average; 17–25 outstanding

^c^ Mean-score instrument: 0 = never … 6 = daily;

^d^ Sum-score instrument: 0–50 very bad learning environment; 51–100 many problems; 101–150 more positive than negative aspects; 151–200 excellent learning environment

^e^ Interquartile range 25–75%

^f^ Pearson’s *r* (effect size Friedman test): *r* = .1 small effect; *r* = .3 medium effect; *r* = .5 large effect

^g^ Cohen’s η (effect size repeated measures ANOVA): η^2^ = .01 small effect; η^2^ = .06 medium effect; η^2^ = .14 large effect

^h^ No post-hoc testing for October 2019. As students had just started their medical study programme, learning environment was not assessed

### Well-being

Well-being was ‘average’ (for detailed sum score ranges, see footnotes, Table 1) (median = 12.0; IQR = 8.0–17.0) when students started their studies in October 2019 and dropped down in December 2019. Here, a median of 9.0 (IQR = 5.0–14.0) expresses the upper limit of ‘below average’. For June 2020, students’ well-being (median = 11.0; IQR = 4.0–15.0) was again ‘average’. In the December 2020, students again reported a well-being score with a median of 12.0 (IQR = 4.0–16.0). Although no overall effect could be identified, the IQRs mirror the rise in heterogeneity of well-being throughout the three semesters of medical school. In June 2020 and December 2020, more students reported a well-being that was ‘severely below average’ (values ≤5.0; Table 1, see footnote).

### Burnout

Emotional exhaustion appeared to be the most affected burnout facet, with an increase from October 2019 (median = 1.33; IQR = 0.67–2.00) to December 2019 (median = 3.0; IQR = 2.0–4.0). In June 2020, emotional exhaustion remained at a comparable level (median = 3.00; IQR = 2.33–4.00) with the IQR expressing emotional exhaustion ‘once a week’ or ‘more often’ for the majority of students. In December 2020, a slight but non-significant reduction in emotional exhaustion was reported (median 2.67; IQR = 1.67–3.67).

The same development but with overall lower medians was observed for personal accomplishment, with a significant rise from October 2019 (median = 0.67; IQR = 0.0–1.33) to December 2019 (median = 1.67; IQR = 1.0–3.0), which slightly intensified in June 2020 (median = 2.0; IQR = 1.0–3.0). IQRs showed that 50% of students ‘sometimes’ up to ‘once a week’ experienced, for example, struggles in solving study-related problems. In December 2020, personal accomplishment showed a non-significant decrease (median = 1.33; IQR = 0.67–2.33).

Depersonalisation also rose significantly in relation to October 2019 but stayed at a low level throughout the first study year (median_Oct 19_ = 0.0; IQR = 0.0–0.0; median_Dec 19_ = 0.33; IQR = 0.0–1.0; median_June 20_ = 0.67; IQR = 0.0–1.67; median_Dec 20_ = 0.33; IQR = 0.0–1.0). The majority of students ‘never’ up to ‘seldom’ experienced, for example, a loss of interest in their studies.

### Learning environment

The learning environment was experienced positively,^2^ both in the ‘old normal’ of December 2019 (mean = 128.2; SD = 21.4) and the ‘new normal’ of June 2020 (mean = 126.2; SD = 22.5). The overall identified effect (η^2^ = .14) is explained by an even improved perceived learning environment in December 2020 (mean = 134.5; SD = 18.2). Mean values express ‘more positive than negative aspects’ for both the in-person and virtual semester.

### Burdens and protective factors

**Fig. 2:**
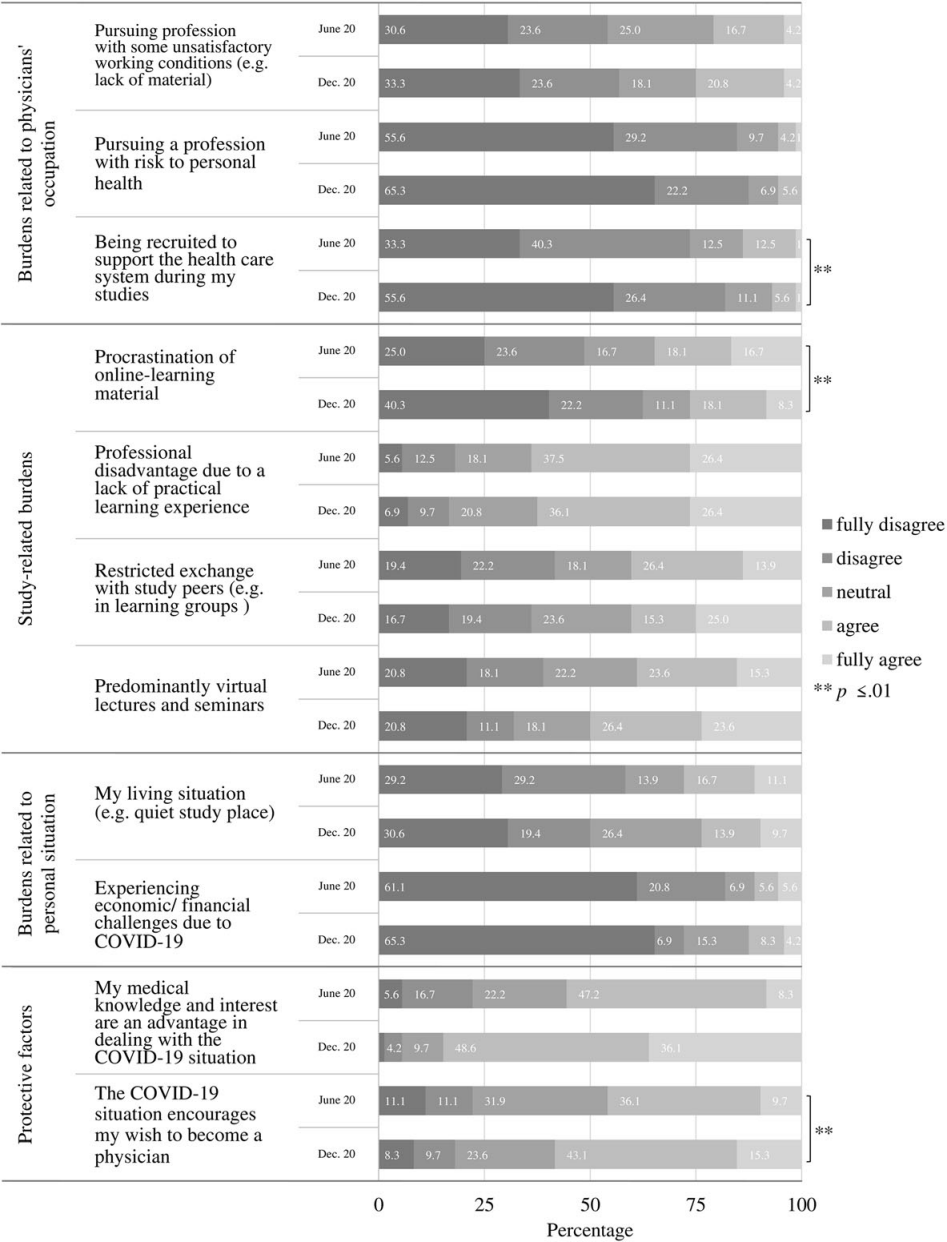
Students’ burdens and protective attitudes in June 2020 and December 2020. Legend: Stacked bar chart of Likert questions (inspired by Choi et al. [17]). The bar charts describe how the first-year medical students answered the five-point Likert scale questions on burdens and protective attitudes. Burdens were categorized as related to physicians’ occupation, study and personal situations. Students were asked ‘Which of the following aspects are a burden to you?’

For a better understanding of the students’ situation during the virtual semesters in June 2020 and December 2020, we asked them about their burdens via single items, which are illustrated in Fig. 2. Datasets from 72 students (90% response rate, female 64%, mean age_study start Oct 2019_ = 20.9, SD = 4.3) were available due to two measurement points only.

Interpreted descriptively in accordance with Fig. 2, for our first-year pre-clinical students, study-related burdens (e.g. restricted exchange with peers) took on greater relevance than burdens related to physicians’ occupation (e.g. unsatisfactory working conditions). Economic burdens were less relevant to the given sample than their current living situation, for example, the presence or lack of a quiet working place. Significantly (medium effect *r* = .33) less students see ‘procrastination of learning online-learning material’ as a burden in the lasting virtual learning situation in December 2020. Also, significantly less students (medium effect *r* = .30) see a potential for their recruitment to the healthcare system during their studies as a burden in December 2020. As illustrated at the bottom of Fig. 2, both in June and December 2020, the majority of students reported their medical interest and medical knowledge as an advantage for dealing with the COVID-19 situation. Additionally, for most students, the wish to become a future physician was encouraged by the COVID-19 situation in June 2020. This increased significantly by December 2020 (large effect *r* = .63).

## Discussion

Our study investigated a cohort of German medical students with regard to their mental health development and perceived learning environment during their first three semesters at medical school. During the longitudinal questioning, which began in October 2019, the COVID-19 pandemic caused a drastic switch to predominantly online study. For the measurement points during this ‘new normal’ (June 2020 and December 2020), we additionally assessed students’ perceived burdens and whether their interest and knowledge in medicine would give them any advantage in dealing with the COVID-19 situation. We discuss our findings considering our sample’s study status and context.

### Students’ mental health and perceived learning environment

Our results reveal a significant increase in depression within the first two months of medical school, which however stayed on the level of ‘mild depression’ until December 2020. The burnout construct of emotional exhaustion was exemplary in this context. Personal accomplishment and depersonalisation—both initially low—experienced severance but stayed within the lower level of the scale. The burnout constructs slightly improved for December 2020 but did not yet have a significant effect. Well-being showed a slight, but overall non-significant drop and recovery for December 2020. Adjustment to the new situation of studying medicine at a university was a greater psychological challenge than the effects of predominant online learning during the COVID-19 pandemic. In evaluating our findings by other studies investigating mental health development for first-year medical students, we identified some challenges elaborated below.

First, in accordance with the meta-analysis by Rotenstein et al. [3]—after 2000, in the European context—there was only a few longitudinal studies matching data at the person-centred level. A survey from the UK assessed students (*n* = 220) longitudinally at the beginning of each academic year in their pre-clinical phase (the first three years). Of these students, 18% reported depression at all measurement points; 5% on more than one occasion and 13% on one occasion [33]. Ludwig et al. [34] questioned a sample of about 300 US medical students in their first and third year on their mental health: 39% of the students fell above the ‘treatment relevant value’ of the applied depression instrument in year 3 (compared to 28% in year 1). The methodological issue in this study was the lack of identifiers to match the data longitudinally on a person-centred level. With regard to the ‘new normal’, a current preprint investigated the depression development of first-year, non-medical US college students before and during the pandemic. A significantly higher PHQ-9 mean of 12.1 was found for April 2020 (during the pandemic) in comparison to a mean of 8.3 in August 2019 (entering college) and a mean of 7.3 in February 2020 (before the pandemic) [35]. Unfortunately, a comparison to our data was restricted as April 2020 was two months before our first ‘new normal’ data (June 2020) were generated.

Second, data on medical students’ mental health are not necessarily reported in consideration of their study status. In the already-cited survey by Dyrbye [2] the sample included students from years 1 to 4, which was subsumed in the status ‘in training’. To allow for prevention instead of treatment [1], we need to identify the root causes and the timing of severance. When entering medical school, 75% of our sample was in a range of 3.0–7.0 for depression measured by the PHQ-9, which defines 10.0 as the cut-off value for ‘treatment relevant’ [19]. In December 2019, the IQR had shifted to 4.0–10.0. Considering the ranges in response behaviour reveals that some students in our sample would have benefited from additional support in their adaption to medical school—by self-care *and* a learning environment favouring mental health. Adapting to the ‘new normal’ was less challenging for the students.

This finding might be explained by the positive development of perceived learning environment as assessed by the Dundee Ready Educational Environment Measure (DREEM). In December 2020, learning environment was rated significantly higher both in comparison to the ‘old normal’ (December 2019) and the previous ‘new normal’ semester (June 2020). Higher DREEM scores are known to associate negatively with psychological distress [32]. Our sample is situated at a newly founded medical faculty and the participants are in the first cohort to experience the curriculum and teaching at this faculty. This situation provokes a certain readiness for adapting to new, unknown (teaching) situations and enforces the partly new conceptualization of learning material. This process was then switched to a predominantly virtual conceptualization. Additionally, students and curriculum designers met in regular virtual meetings to exchange information on the situation of exclusive virtual learning.^3^ The power of such ‘relationship-centred education’ [36] was emphasized by Wald [14] in his advice on how to cultivate resilient learning environments during a crisis, such as the COVID-19 pandemic. Also, our students had known each other from the in-person semester and might had built some substantial relationships. Of course, a positively perceived learning environment is just one explanatory puzzle piece. E.g., a current US-based study found that first-year students reporting an application of the adaptive coping strategy of positive reinterpretation and growth (e.g. making the best of the situation by growing from it) could reduce their burnout facet personal accomplishment by 60% [37]. The study does not include a longitudinal perspective on burnout or coping strategy development within the first year.

Based on the discussion of our findings, we argue for the necessity of a) assessing the same cohort in the continuity of their studies; b) treating measurement points as a dependent sample; c) specifying the sample’s study status; and d) investigating study behaviour (e.g. coping strategies) and context (e.g. learning environment).

### Students’ burdens and protective attitudes

To better understand the ‘new normal’ situation of our students—which brought drastic changes [8]—we evaluated their burdens and protective attitudes. Interpreted descriptively, students’ burdens are rather dominated by study-related aspects in comparison to occupation-related aspects. The last-mentioned are still in the future for first-year students and, because of their yet restricted clinical experience, less relevant. Additionally, until June 2020, Germany had not experienced severe case fatality rates [38], triage scenarios or overwhelmed intensive care units [39]. In June 2020, the complete lockdown was over. GermanTrend representative questioning [40] revealed a perceived reduction in infection risk, an agreement to loosen the restrictions and an economic comfort zone for the majority of the population in June 2020. These facts might have caused a positive student response bias.

In December 2020, Germany was in a lockdown ‘light’ situation, after a relatively ‘old normal’ summer. In the GermanTrend questioning of December 2020 [41], the majority of the 18–39 age group agreed with the political steps to control the spread of the pandemic. With regard to our occupation-related items, less students agreed that they were worried about being recruited to support the healthcare system. Students had learned that this had not happened so far, especially in their role as pre-clinical students. Additionally, in December 2020 the media’s slant—after 9 months in the pandemic situation—had severely changed. During the first months of the pandemic and with very many unknowns, a greater attention might have been paid to maximally supporting the healthcare system, whereas over time, debates about lockdown and re-opening dominated.

As described in the background to our study, in a survey for 1800 students in various faculties at German universities, digital learning was shown to be satisfactory to the majority of these students with computer access, self-reported digital skills and information-sharing behaviour. In the referred study, a cluster analysis revealed better equipped and more highly skilled students were less prone to perceived stress [18]. The absence of economic burdens (assessed by the item ‘The following aspect was a burden for me: Experiencing economic/financial challenges due to COVID-19’) in our sample could suggest sufficient digital equipment—an interpretation to be made with caution. Additionally, familiarity among our small student cohort might have been an advantage in the continuity of information-sharing behaviour. Händel et al. [18] found that their cluster of ‘digital-ready’ students —who reported less tension, overload, worries and loneliness— had a more intense social (technology-based) interaction behaviour. Again, this needs to be regarded as a possible interpretation for our sample—however, without an empirical data foundation. Our students rated their knowledge and interest in medicine as a ‘protective’ factor. In a questionnaire addressing Belgian medical students in their final year, the majority of students agreed that the pandemic crisis had increased the theoretical knowledge that they would carry forward [42]. Additionally, our students’ perceived encouragement for their chosen future profession had intensified significantly by December 2020. This answer might have been biased by the fact students had grown into their study programme and had developed a more profound idea of their future occupation. Also, students might have perceived a solid German management of the pandemic until December 2020 and experienced some self-satisfaction at the prospect of serving a comparatively substantial healthcare system in the future. As stated by Wald [14], a recognition of individuals’ contributions in a crisis—also a prospective in the professional lifecycle—can matter.

### Limitations and the need for critical follow-up

Our findings regarding the ‘new normal’ only apply to their time of assessment in June 2020 and December 2020. In June 2020, our sample’s ‘new normal’ of virtual learning might have been dominated by excitement for the ‘new’, enjoyment of more study-related autonomy and less commuting stressors. The December 2020 measurement point was before a continuing lockdown situation, which currently (April 2021), does not offer the prospective for re-exposing students to campus-based learning. The next questioning will take place in June 2021. We do not yet know any of the effects of the continuity of restricted campus-based learning and challenges of re-engagement—especially for a cohort of students, which is currently more used to virtual learning than in-person learning. Ferrel and Ryan ([13], p. 2) stated the importance of being aware of ‘challenges to re-engage students within the community spirit of medical school once restrictions are lifted’.

## Conclusion

From our findings, we conclude that: 1) the ‘new’ when entering medical school has the highest change impact on our students’ mental health; 2) the ‘new normal’ of predominantly online-learning did not cause any additional severance; 3) the readiness for change in the context of a newly designed study programme might have been beneficial with regard to students’ positively perceived learning environment during the virtual semesters; 4) for a cohort of first-year students, burdens in the ‘new normal’ semester are more study related (e.g. restricted peer exchange) than occupation related (e.g. being recruited to support the healthcare system); and 5) caring for medical students’ mental health and monitoring it through person-centred and longitudinal means should be a concern regardless of the pandemic.

## Data Availability

The datasets generated and/or analysed during the current study are not publicly available for reasons of data protection and data storage restricted to university internal servers but are available from the corresponding author on reasonable request.

## Footnotes

1 MFT Medizinischer Fakultätentag der Bundesrepublik Deutschland e. V. (2015). Nationaler Kompetenzbasierter Lernzielkatalog (NKLM). Retrieved from http://www.nklm.de/files/nklm_final_2015-07-03.pdf

2 For an overview of the Dundee Ready Educational Environment Measure (DREEM) results of international medical study programmes, see [31]: Average DREEM results mean = 120.0 (max. Sum-score = 200); DREEM results in the newly developed, reform-based study programmes mean = 128.4. This is confirmed by Chan et al.’s [32] review on the application of DREEM citing findings on higher DREEM scores in study programmes with a more modern curriculum.

3 This personal talk took place on 6 Oct. 2020 with the curriculum design team leader (unreferenced)
